# Parental Factors Associated with Mexican American Adolescent Alcohol Use

**DOI:** 10.1155/2013/205189

**Published:** 2013-03-14

**Authors:** Cristina Mogro-Wilson

**Affiliations:** School of Social Work, University of Connecticut, 1798 Asylum Avenue, West Hartford, CT 06117-2698, USA

## Abstract

The purpose of this study is to further the understanding of how parenting and the relationship between the parent and the youth influence adolescent alcohol use in Mexican American families, with particular attention to acculturation. Results indicated that parental warmth is a strong factor in predicting adolescent alcohol use among Mexican adolescents. The parent-youth relationship played an important role in lowering alcohol use for Mexican American youth. Acculturation has an impact on the level of warmth, control, and the parent-youth relationship for Mexican American families. Findings indicate that there are unique family mechanisms for Mexican American families that should be considered when developing prevention and treatment options.

## 1. Introduction

Latinos constitute the largest and most rapidly growing ethnic group in the US [1]. Currently and over the past twenty years, non-Latino adolescent alcohol use has declined, yet alcohol use among Latino youth has remained high [2]. The Latino population continues to grow and is at a high risk because of the trends in demographics. Mexican Americans constitute 67% of the Latino population, or approximately 28 million individuals [3]. Latino youths have a higher high school dropout rate, a higher proportion of families living in poverty, and the highest fertility rate compared to other minority groups [1]. In addition, there are numerous alcohol-related problems reported by young drinkers, such as interpersonal problems, impaired school and work performance, risky sexual behaviors, and drunk driving [4–6]. For many reasons, such as limited access to the population and an increased diversity of the Latino population, research on Latino adolescent alcohol use is sparse and many studies group all Latino subgroups together, making it difficult to understand differences and similarities between groups [7, 8]. Study findings on a variety of health outcomes, including substance use, have shown differences by Latino subgroup [9, 10]. This study focuses on Mexican American youth because of the lack of specific knowledge on parenting in this subgroup of Latinos. In addition, various studies have shown that Mexican Americans, compared to other Latino subgroups such as Puerto Rican's, have unique family composition, cultural attitudes, and substance use [11, 12]. It has been demonstrated that family mechanisms, in particular parenting styles, may be of important influence on substance use tendencies among young individuals. 

Baumrind's [13] theoretical framework of parenting delineated four dimensions to parenting styles: permissive, authoritarian, authoritative, and uninvolved. One of the critical ideas from Baumrind's four quadrants of parenting styles is that parenting revolves around issues of warmth and control. The categorization of these two characteristics, warmth and control, creates a typology of four parenting styles. *Permissive* parents are nondirective and are lenient and are warm and loving; *authoritarian *parenting is associated with low parental warmth and stricter rules; *authoritative* parenting is associated with high parental warmth and clear limits that are negotiated; *uninvolved* parent scan be rejecting and neglectful [14, 15]. 

Baumrind's [13] seminal studies showed that authoritative parenting, or warm and firm parenting, has higher levels of adolescent competence and psychosocial maturity than their peers who were raised by parents who were permissive, authoritarian, or uninvolved parents. Dozens of studies over the past fifteen years that all used different methods, samples, and measures reached the same conclusion that authoritative parenting is associated with advantages in adjustment, school performance, and psychosocial maturity [16, 17]. Research on parenting styles and alcohol use among non-Latinos indicates that authoritative parenting is associated with less alcohol use [18, 19]. The majority of research on parenting and adolescent outcomes has been conducted with European American families, and increasingly with families of color [20]. There has been surprisingly little empirical research on the role of parenting and the role it plays for alcohol use in Mexican American adolescents. In light of this, this paper will investigate the connection between parenting and alcohol use for Mexican American youth.

The literature characterizes Mexican American families as having strong loyalty and closeness to the extended family (*familismo*), interdependent relationships among different generations, and a hierarchical family structure with clear expectations for parent and child roles. This type of family structure is often characterized as authoritarian based on the strong emphasis on parental respect (*respeto*) and authority [14, 21, 22]. In addition to the hierarchical family structure where parents have authority [23, 24], the parent-youth relationships are also informed by cultural norms of *personalismo* and *simpatia*, which place an emphasis on warm personal relationships [23]. This dual cultural emphasis on warmth and control is consistent with an authoritative style. Some literature has also described Mexican American parents as relaxed and permissive toward their children, which has been interpreted as an acceptance of the adolescents' individuality [23, 25]. Parenting is embedded in the culture of a group, and in an effort to understand parenting, the cultural context must be considered.

Acculturation is the social and psychological influences that occur due to continuous contact between individuals from different cultures [26]. Parental acculturation has a strong influence on the adolescents' development [27]. Vega et al. [28] found that composite measures of adolescent and parent acculturation are better predictors of alcohol use than the gaps between adolescent and parent acculturation. Parenting styles may be more fluid than what the traditional cultural norms suggests and depend on parents' adherence to traditional values, acculturation level, and the larger context of their lives [22]. Within the cultural context, parenting practices among Mexican American families can range in a variety of ways, and it is not entirely clear how acculturation relates to parenting styles. 

The parent-youth relationship is another important dynamic to consider when looking at family mechanisms, particularly in Latino families. The interactions, behaviors, and emotions exchanged between parents and their adolescents can be warm or hostile. The type of parenting style used is often a reflection of how the parents were raised. However, the parent-youth relationship is a unique set of interactions that has been linked to adolescent problem-solving behaviors and feelings of being able to control events that can affect him or her [29, 30]. The protective influences of Latino family centeredness and familismo include support, counseling, advice giving, and modeling of behaviors. The support and advice giving in a Latino family builds a relationship between the youth and parent that is above and beyond the typical parenting style. The high quality of parent-youth relationships has been linked to the positive development of adolescents in multiple domains such as depressive symptoms, aggression, and substance use [31, 32]. Mexican American families are often considered to be highly child-centered, with parent-youth relationships often viewed as more important than the marital relationship [23]. The meaning and influence of parenting practices and the parent-youth relationship may differ across ethnic groups. Parental practices are often shaped by culture-specific norms and by ecological factors, such as the process of acculturation [33].

There have been few investigations on the relationship of parenting style to adolescent alcohol use among Mexican American youth specifically. A national sample on Latino adolescents utilizing the Add Health data indicated that high amounts of parental warmth, control, and parent-youth relationship decreased alcohol use [34]. However, acculturation of the parent (parent place of birth) did not influence parenting and the study did not investigate the findings for Mexican American youth [34]. A study of alcohol and other drug use among adolescents found that a positive relationship with the father was associated with less use of alcohol among the Latino subsample. Parental control or strictness was associated with lower substance use [35]. Another study of Latino preadolescents found lower rates of smoking initiation among youth who reported higher levels of parental monitoring and communication about problems with parents [36]. Research that included a subsample of Latino youth of approximately ages between 11 and 13 found that parental monitoring was associated with adolescents' lower use of drugs [37].

Thus, while parental control and warmth have been associated with less drug use, the research that was conducted thus far with Latino youth has several key limitations. First, research has been conducted with Latino samples that reflect substantial diversity with respect to culture, historical context, and history in the US This paper will address this gap by investigating Mexican American families. Second, the literature has not examined the relationship of acculturation level and the influence it may have on a parenting style and alcohol use for Mexican American families. Third, there has been little examination of the independent and combined influence of the role that the parent-youth relationship plays in relationship to parenting and its connection to alcohol use. The present study addresses these gaps in its investigation of the relationship between Mexican American parenting style, the parent-youth relationship, and adolescent alcohol use while taking into consideration the parents' acculturation level. Based on prior theoretical and empirical work, the study is guided by the following hypotheses.

H1: Mexican American parents who are high in control and those parents who are low in warmth (authoritarian parenting) will have adolescents who use alcohol less compared to those with high amounts of control and high warmth (authoritative parenting). The hypothesis is that Mexican American adolescents respond better to authoritarian parenting, as this is a traditional cultural norm.
H2: Those parents using high warmth will have a positive parent-youth relationship. Those parents who have a favorable view of their relationship with their adolescent will have adolescents who use alcohol less.
H3: Less acculturated parents use authoritarian parenting (more controlling). More acculturated parents use authoritative parenting (less controlling).
H4: Acculturated youth and families will have high levels of alcohol use, low levels of control, high levels of warmth, and a good parent-youth relationship.

## 2. Methodology

The data used for this paper is from the National Longitudinal Study of Adolescent Health (Add Health) based in the University of North Carolina at Chapel Hill. Add Health is a school based, longitudinal study of the health-related behaviors of adolescents and their outcomes in young adulthood. Add Health uses a clustered sampling design that is school-based so that the school is the initial point of contact between the researchers and the respondents. There are 132 schools in the core study. A self-administered questionnaire was taken in schools between September 1994 and April 1995 during a class period for grades 7–12. All of these students (83,105) were used as a sampling frame to identify a stratified (by grade and gender) random sample of 16,044 adolescents [38]. These 16,044 students comprise the core sample and were used for in-home interviews. All data used in this paper is taken from the in-home interview [39]. A parent, usually the mother, also completed a questionnaire. Ninety-five percent of the respondents for the in-home interview were female head of households, 88% of which were the biological mother; the remaining were grandmothers, step mothers, or aunts. One year later, the Wave 2 in-home sample was composed of adolescents who participated in the first wave of the in-home component and resulted in 10,547 participants [39]. The response rate for Wave 1 is 79%, and the response rate for Wave 2 is 88% [40]. For the purpose of this study only the adolescents who responded that they were Mexican American and had data on alcohol use behaviors for Waves 1 and 2 were used for this sample *n* = 956.

### 2.1. Measures

#### 2.1.1. Alcohol Use

Adolescents were asked about how often they consume alcohol and how often they get drunk in the past 12 months, responses ranged from almost every day, three to five times a week, one or two days a week, two or three days a month, once a month or less or one or two days in the past year, or never. The drinking and getting drunk questions were asked in Wave 1 and Wave 2. 

#### 2.1.2. Parent Acculturation

Parents were asked if they were born in the United States. 

#### 2.1.3. Adolescent Acculturation

The adolescent was asked if they were born in the US.

#### 2.1.4. Family Acculturation

The adolescent was asked what language they usually speak in their home. 

#### 2.1.5. Parental Control

Youth were asked if their parents allow them to make their own decisions about “(a) the time you must be home on weekend nights? (b) the people you hang around with? (c) what you wear? (d) how much television you watch? (e) which television programs you watch? (f) what time you got to bed on week nights? (g) what you eat?” A scale was created where the sum of the 7 questions was divided by 7, then multiplied by 100, giving a percentage. Those with a high percentage are highly controlled. 

#### 2.1.6. Parental Warmth

Youth were asked how warm and loving their mother/father was towards them options ranged 5 = strongly agree, 4 = agree, 3 = neither agree nor disagree, 2 = disagree, 1 = strongly disagree, where high numbers indicate high warmth. Mother and father warmth were averaged together with a Pearson correlation of .151, *P* < .001.

#### 2.1.7. Parent-Youth Relationship

Parents were asked: “How often would it be true for you to make each of the following statements about your child” Indicator no. 1: you get along well with him/her; Indicator no. 2: you make decisions about his/her life together; Indicator no. 3: you feel you can really trust him/her. This was measured using a 5-point scale starting at always, often, sometimes, seldom, and never. Where higher numbers indicate a better relationship. 

#### 2.1.8. Peer Alcohol Use

Youth were asked how many of their three best friends drink alcohol at least once a month, and responses ranged from 0 to 3, peer alcohol use was used as covariate.

#### 2.1.9. Income

Income was measured by a question in the parent questionnaire that asked about total income before taxes for everyone in the household, income was used as a covariate.

## 3. Results

The study sample consisted of 956 Mexican American adolescents (*n* = 956). Parental acculturation level that was measured by parent place of birth indicated that almost half of the parents were born inside the US (46%) with 54% born outside of the US Most of the Mexican American adolescents were born in the US (83%). About half of the adolescents spoke primarily English in the home (56%), indicating that 44% of the sample spoke primarily Spanish in the home. Gender was divided almost equally among adolescents with slightly more females with 52% and males with 48%. The adolescents in the sample ranged from 7th grade to 12th grade at Wave 1. Twelve percent of the sample were in the seventh grade, 12% in 8th grade, 13% in 9th grade, 24% in 10th grade, 19% in 11th grade, and 18% in 12th grade, and 3% of the sample refused to answer or was not in a school that had traditional grade levels. Income levels indicated that 64% of the families earned less than $34,000 annually (Table 1). 

Parental warmth measured on a scale of 1 to 5 ranging from very low warmth to very high warmth had mostly parents indicating very high or high warmth (63%) and the rest (37%) indicting very low, low, or average warmth. Adolescents indicated that parental control on a scale of 0 to 100 that 18% were not controlling, 62% were slightly controlling, and 19% were very controlling with only 2% being totally controlling (Table 2).

The parent-youth relationship was measured by three different variables. The first, get along well together (py1), found most parents saying often or always (88%) with the remaining indicating never, seldom, or sometimes (12%). The second parent-youth relationship variable (py2), asking parents if they make decisions together with their adolescent, found that 70% often or always make decisions together and 30% never, seldom, or sometimes make decisions together. The third parent-youth relationship variable (py3) asked the parents if they trust their adolescent and found that 85% always or often trust them and 15% never, seldom, or sometimes trust them (Table 2).

### 3.1. Structural Equation Modeling Analysis

Structural equation modeling (SEM) is used for an analysis of the effects between identified independent variables. The model for this analysis will be based in the existing research and theory. The data was analyzed using Mplus and the appropriate sample weights created by the AddHealth staff [38, 41, 42]. 

Multivariate normality was evaluated using Mardia's index. The *P* value for the multivariate index was statistically significant (*P* < 0.05). Examination of univariate indices of skewness and kurtosis revealed only two variables with absolute skewness values and kurtosis values above 2.0, the getting drunk variables from Wave 1 and Wave 2. Given the presence of nonnormality the Mplus analysis utilizing sample weights, a complex analysis was used using MLR, maximum likelihood estimation with robust standard errors, which is robust to nonnormality. 

**Figure 1:**
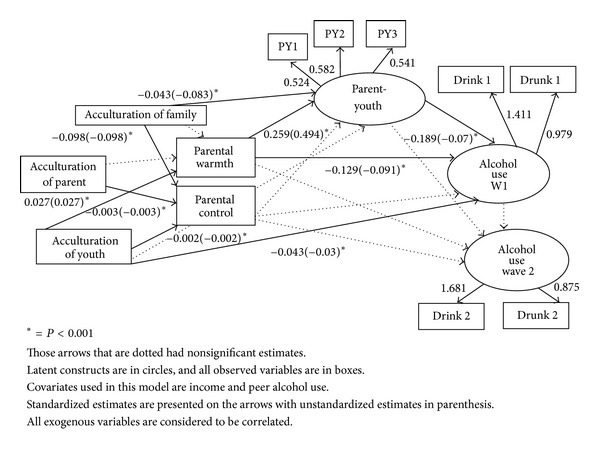
SEM model for explaining the relationship between parenting style, acculturation, and alcohol use in Mexican American adolescents.

A variety of indices of model fit were evaluated resulting in good model fit. The Bollen-Stine bootstrapped chi-square test yielded a value of 88.665, with degrees of freedom of 36, and a *P* value of 0.001. The Root Mean Square Error of Approximation (RMSEA) was 0.039, which was less than 0.08 indicating good model fit. The *P* value for the test of close fit was 0.958, which was nonsignificant indicating good model fit [43]. The test of close fit provides a one-sided test of the null hypothesis that the RMSEA equals .05, and this is what is called a close-fitting model. The Comparative Fit Index was 0.986 which was higher than 0.95, indicating again good model fit [44]. The Tucker-Lewis Index (TLI) was 0.97, indicating a very good fit. The indices uniformly point towards good model fit. Inspection of the residuals and modification indices revealed no significant points of ill fit in the model.  Figure 1 presents the parameter estimates incorporating sampling weights. The residuals are in standardized form and are reflective of unexplained variance in the endogenous variables. 

Path coefficients for parent place of birth affecting parental control did result in statistical significant coefficients for Mexican American youth. On average, for parents born inside the US, parental control would decrease by .003 for Mexican American youth (*P* < .001) compared to those parents born outside the U.S. On average if English was spoken at home, parental control would decrease by .098 compared to those who spoke Spanish at home (*P* < .001). If English was spoken at home, the parent-youth relationship decreases by .043 compared to if Spanish was spoken in the home (*P* < .001). If the adolescent was born in the US, alcohol use at Wave 1 increased by .043 compared to those who were born outside the US (*P* < .001). If the adolescent was born in the US parental control decreased by .002 compared to if the adolescent was born outside the US (*P* < .001). If they were born in the US parental warmth increased by .027 compared to if the adolescent was born outside the US (*P* < .001). 

For every one unit increase in parental warmth, the parent-youth relationship increases by 0.259 (*P* < .001). Parental control on the parent-youth relationship did not result in statistically significant coefficients. For every one unit increase in the parent-youth relationship, adolescent alcohol use is 0.189 units lower for Mexican American youth (*P* < .001). A one unit increase in parental warmth results in alcohol use at Wave 1 decreasing by 0.129 (*P* < .001). For Mexican American youth, parental control does not significantly influence the use of alcohol. 

The covariate estimates for presentation purposes are not on the figure. For Mexican American youth, every additional friend who uses alcohol, parental control decreases by .007 (*P* < .001). For every additional friend who uses alcohol, alcohol use in Wave 1 increased by .678 (*P* < .001). Income did significantly influence parental warmth for Mexican American youth; for every one thousand dollar increase in income, parental warmth increased by 5.314 (*P* < .001). For every one thousand dollar increase in income, parental control decreased by .098 (*P* < .001). 

## 4. Discussion

The results of this study contribute to the understanding of how family mechanisms, specifically parenting and the parent-youth relationship, influence the use of alcohol for Mexican American adolescents and the impact of acculturation on these factors. Few studies are able to investigate within Latino subgroups. However, these subgroups are often very different in their cultural values and in their decision to use substances [7]. For these reasons, the model was tested for Mexican American youth. The dependent variable of alcohol use was measured once at Wave 1 and again one year later at Wave 2. None of the variables used in the model significantly predicted alcohol use at Wave 2. In this model, there is no prediction of change or no activity with intent to have an impact one year later. This may be due to the short-time period of one year from Wave 1 to Wave 2, and therefore sustained changes may have had difficulty becoming apparent. Therefore, all findings are relational and not causal.

One of the most interesting findings for Mexican American adolescents was the influence of acculturation on family mechanisms. Mexican American parents who are more acculturated, or born inside the US, and those who spoke English in the home had lower levels of parental control compared to less acculturated parents. This relationship was expected, given that if the parent is more acculturated their parenting style would become more similar to non-Latinos, and there would be less parental control. For Mexican American youth, if the adolescent was born in the US, there was more parental warmth and less control, compared to less acculturated youth. This finding confirms the idea that acculturation does have influence parenting for Mexican American families. Interestingly, a previous model testing a Latino sample showed that there was no relationship between parental acculturation and parental control [34]. The finding for Mexican American families indicates the increased influence of acculturation on parenting that does not exist in other studies looking at other subgroups of Latinos. This strengthens the rationale to continue to do research on subgroups of Latinos, as acculturation is creating differential effects on behaviors and outcomes. Various reasons for this difference in the level of acculturation on parenting necessitate further investigation, including the community characteristics and a more in-depth examination of the acculturation process for the parent, youth, and family. 

Language spoken at home, a measure of family acculturation level, also had an influence on the parent-youth relationship. If English was spoken in the home, there was a decrease in the parent-youth relationship. This could possibly be contributed to a widening gap between the parent and youth as distance is created from the original culture. Traditional Latino families may have a stronger connection with their youth, and as the family moves toward being more acculturated and speaking more English in the home, the parent and the youth relationship suffers. Previous model testing did not show this relationship for a Latino sample [34]. The influence of language on the parent-youth relationship and the adolescent place of birth impact on parental warmth and control are unique to Mexican American families. 

There have been very few research studies that show the impact of acculturation on parenting style. However, there is contradictory evidence. Some studies show that the gap between the child and parent acculturation levels lead to more alcohol use, while others have found that the overall level of parent and child acculturation determines alcohol use [28, 45]. Martinez [46] found that family acculturation level is a better construct rather than differences in parent and adolescent acculturation when predicting substance use. This model does not support the finding of Martinez [46] where acculturation of the youth is the only direct predictor of alcohol use for adolescents. 

Mexican American family mechanisms are influenced by acculturation factors. Similar to Delva et al. [7], who found that acculturation level was measured by preferred language spoken, Mexican adolescents were more likely to use alcohol or marijuana. Acculturation has an impact on level of warmth, control, and the parent-youth relationship for Mexican American families. 

Although there is an effect of parent place of birth on parental control, parental control does not have a significant effect on alcohol use. However, it did have an effect for the entire Latino sample [34]. The large Latino sample (*n* = 1887) consisted of 51% Mexican American, 30% other subgroups such as Cubans and South Americans, and 19% Puerto Rican youth. The Mexican American subgroup could be pulling down the covariate estimate while the other subgroups in the Latino group are pulling the covariate up. This suggests differences among Latino subgroups around the understanding of parental control in relation to alcohol use. It is possible that there are cultural differences for the way parental control is practiced or the way that adolescents perceive parental control. The differences with the Mexican American youth compared to a Latino sample in Mogro-Wilson's [34] can be explained by the other subgroups not analyzed. 

Parenting in a warm and loving manner, as interpreted by youth, was related to a decrease in alcohol use for Mexican-American youth. In addition the more warmth and love showed to the youth was also related to a better parent-youth relationship which decreases alcohol use. The importance of warmth found in this Mexican American sample is consistent with cultural norms that stress nurturing of the child in the context of a respect and a strong family [23, 34, 47]. The present study is unique in providing evidence for the role of the parent-youth relationship and the role of acculturation on warmth.

This research indicated that the stronger the parent-youth relationship Mexican American youth used less alcohol. The parent-youth relationship is an important construct in family mechanisms that can have an impact on alcohol use beyond typical parenting constructs of warmth and control. This finding highlights the importance of the parent-youth relationship in the use of alcohol and indicates the importance to target this construct in interventions and prevention. Parenting interventions should include aspects of building and strengthening parent-youth relationships, activities to foster the growth of the relationship, make decisions together, and build trust.

### 4.1. Limitations

Limitations of the study include the age of the data; the data for this study was collected in 1995; however, there has been little change in alcohol use over time for this population. Comparing the 1995 Add Health data to national alcohol use rates based on the Monitoring the Future Study, the rates used in the present study are similar to the national use rates [48]. The Monitoring the Future Study national use rates for Hispanic youth show in 2009 when asked if they have ever used alcohol, 19% of eighth graders responded that they had used alcohol, and 34% of tenth graders and 40% of twelfth graders indicated they had drank in the past year [10]. The Add Health sample of Mexican American adolescents used in this study indicates more alcohol usage with 35% of 8th graders reported using alcohol, 53% of tenth graders and 57% of twelfth graders. In addition to changes in alcohol use over time, there may also be changes in family dynamics that occur over time that are impossible to predict, and this adds to the limitations of the present study. 

In addition to the date of data collection, there are measurement issues such as the construct of parental control. The measure used for parental control was unable to indicate negotiation, an important aspect of parenting, and this limits the findings. In addition, the parent-youth relationship would have benefited from a well-validated questionnaire such as the Alabama Parenting questionnaire or the Egna Minnen av Barndoms Uppfostran—My Memories of Upbringing (EMBU) [49, 50].

A limitation to the acculturation findings relates to how acculturation was measured. Measuring acculturation by language spoken at home and how long the individual has been in the country still holds validity. Many researchers continue to conceptualize and measure individual acculturation in a unilinear way [51, 52]. The variety of acculturation measures also shows that there is no consistent way to measure acculturation in the field. For these reasons and due to constraints of the secondary data set, acculturation was measured by place of birth and language spoken at home. However, this is a unidimensional way to measure acculturation, and the use of better measures of acculturation should be used in the future to see if the results are similar. Studies have demonstrated that proxy acculturation items, such as place of birth and language spoken at home, can be useful to assess acculturation in situations where use of a more comprehensive acculturation scale is impractical [52]. Language is considered the strongest single predictor of acculturation [53].

## 5. Conclusion

This study confirms the importance of the family as a protective factor for alcohol use in adolescence for Mexican Americans. Parenting and the relationship the parent has with their youth influences an adolescent's choice to use alcohol. The role of parents to reduce the risk taking behavior of alcohol use is a strong finding. The present study used combined scores of parental warmth and control of the mother and father; however, separating these differences to see what kind of contribution the mother versus the father in the role of parenting would be valuable. Extended research on other subgroups of Latinos would be useful in understanding the similarities and differences between Mexican American and other subgroups, such as Puerto Ricans. Further qualitative research would be useful in describing the parenting styles and practices in diverse groups of Mexican American families. It would be valuable to examine if parenting characterized by warmth is viewed as more consistent with Mexican American cultural norms. Further research that measures acculturation bidimensionally and its influence on parenting and the parent-youth relationship would prove useful to the understanding of alcohol use. 

Parental warmth can function as a protective factor for Mexican American families in preventing alcohol use. However, this is in the context of the parent-youth relationship, which is a necessary component to the model. Common beliefs about Latino families characterize parenting as strict, controlling, and abrasive. This model found no relationship between parental control and alcohol use for Mexican American families. Parental warmth plays a large role in adolescent alcohol use, as warmth increases the parent-youth relationship improves and alcohol use decreases. This paper supports the idea that Mexican American families have a protective quality of high warmth and a good parent-youth relationship, and as they acculturate the parent-youth relationship decreases and alcohol use increases.

Few interventions to prevent underage drinking have specifically targeted Latino youth or families [54, 55]. Further research is needed to design culturally appropriate interventions that are likely to be accepted among Latino families. Programs designed to improve parental warmth and caring behaviors toward the youth and programs to promote positive parent-youth relationships are likely to reduce adolescent alcohol use. Interventions that focus on relationship building, across the acculturation divide between the youth and parent, could help promote a positive parent-youth relationship. In addition focusing on trust building activities and making joint decisions between the parent and the youth could provide a protective factor from using alcohol in adolescence. 

## Figures and Tables

**Table 1:** Descriptive characteristics of Mexican American youth (*n* = 956).

Characteristic	Frequency	(%)
Place of birth: parent		
US	440	46%
Outside the US	516	54%
Place of birth: adolescent		
US	792	83%
Outside the U.S.	164	17%
Language Spoken at home		
English	538	56%
Spanish	418	44%
Sex		
Female	494	52%
Male	462	48%
Grade (at Wave 1)		
7th	112	12%
8th	114	12%
9th	121	13%
10th	230	24%
11th	180	19%
12th	167	18%
Not in school	1	0%
Refused	8	1%
School does not have grade levels of this kind	23	2%
Income		
$1,000–24,000	457	48%
$25,000–34,000	153	16%
$35,000–44,000	116	12%
$45,000–54,000	96	10%
$55,000–64,000	60	6%
$65,000–74,000	27	3%
$75,000–84,000	25	3%
Over $85,000	22	2%

**Table 2:** Parenting characteristics of Mexican American families (*n* = 956).

Parenting characteristic	Frequency	(%)
Parental warmth		
Very low warmth	28	3%
Low warmth	186	19%
Average warmth	142	15%
High warmth	296	31%
Very high warmth	304	32%
Parental control		
Not controlling	168	18%
Slightly controlling	595	62%
Very controlling	178	19%
Totally controlling	15	2%
Parent-youth relationship		
Get along well (PY1)		
Never	3	0.3%
Seldom	13	1%
Sometimes	101	11%
Often	314	33%
Always	525	55%
Make decisions together (PY2)		
Never	23	2%
Seldom	38	4%
Sometimes	230	24%
Often	316	33%
Always	349	37%
Trust them (PY3)		
Never	7	1%
Seldom	25	3%
Sometimes	117	12%
Often	218	23%
Always	589	62%
